# Inulin-type fructans supplementation improves glycemic control for the prediabetes and type 2 diabetes populations: results from a GRADE-assessed systematic review and dose–response meta-analysis of 33 randomized controlled trials

**DOI:** 10.1186/s12967-019-02159-0

**Published:** 2019-12-05

**Authors:** Long Wang, Hong Yang, Hao Huang, Chao Zhang, Hong-Xia Zuo, Pan Xu, Yu-Ming Niu, Shi-Shi Wu

**Affiliations:** 1 Center for Evidence-Based Medicine and Clinical Research, Taihe Hospital, Hubei University of Medicine, No. 32, South Renmin Road, Shiyan, 442000 China; 2 Department of Histology and Embryology, School of Basic Medical Sciences, Hubei University of Medicine, No. 30, South Renmin Road, Shiyan, 442000 China; 3 Hubei Key Laboratory of Embryonic Stem Cell Research, Taihe Hospital, Hubei University of Medicine, Shiyan, 442000 China

**Keywords:** Inulin-type fructans supplementation, Glycemic control, Type 2 diabetes, Prediabetes, Meta-analysis

## Abstract

**Background:**

Currently, many clinical trials have shown that inulin-type fructans (ITF) supplementation is associated with glycemic control; nevertheless, the results are inconclusive. The aim of this meta-analysis of randomized controlled trials was to assess the effects of ITF supplementation on glycemic control.

**Methods:**

PubMed, EMBASE and the Cochrane Library were searched for eligible articles up to March 6, 2019. A random-effects model was used to analyze the pooled results, and the Grading of Recommendations Assessment, Development, and Evaluation (GRADE) system was applied to assess the quality of evidence. The dose–response model was used to recommend the daily dose and duration for ITF supplementation.

**Results:**

Thirty-three trials involving 1346 participants were included. Overall, ITF supplementation could significantly reduce concentrations of fasting blood glucose (FBG), glycosylated hemoglobin (HbA1c), fasting insulin (FINS) and homeostasis model assessment-insulin resistance (HOMA-IR). In the prediabetes and type 2 diabetes (T2DM) population, a more significant reduction in FBG [weighted mean difference (WMD): − 0.60 mmol/l; 95% CI − 0.71, − 0.48 mmol/l; high rate], HbA1c (WMD: − 0.58%; 95% CI − 0.83, − 0.32%; high rate), FINS (WMD: − 1.75 µU/ml; 95% CI − 2.87, − 0.63 µU/ml; low rate), and HOMA-IR (WMD: − 0.69; 95% CI − 1.10, − 0.28; low rate) were observed, and ITF supplementation with a daily dose of 10 g for a duration of 6 weeks and longer was recommended. Moreover, subgroup analyses suggested that the effects of glycemic control were significantly influenced by the sex of the subjects and the type and the method of intake of ITF.

**Conclusions:**

Our analyses confirmed that these four main glycemic indicators were significantly reduced by ITF supplementation, particularly in the prediabetes and T2DM population. Evidence supports that reasonable administration of ITF supplementation may have potential clinical value as an adjuvant therapy for prediabetes and T2DM management.

*Trial registration* The trial was registered at PROSPERO as CRD42018115875 on November 23, 2018.

## Background

Recently, abnormal blood glucose metabolism is experiencing rapid growth around the world, and there are more than 463 million adults with diabetes and an additional 374 million adults with impaired glucose tolerance worldwide [1]. Studies have shown that abnormal blood glucose is related to the development and prognosis of some chronic diseases, including type 2 diabetes (T2DM) and cardiovascular diseases [2–4]; thus, glycemic control is necessary. Except for regulatory control by hypoglycemic agents, dietary regulation and lifestyle modification have also been reported to be effective in glycemic control [5]. Studies have shown that intake of some dietary supplements, including omega-3 fatty acids [6], zinc [7] and coffee [8], could enhance glycemic control and reduce the risk of diabetes and its related complications. Moreover, the epidemiological data in some relevant studies suggest an association between glycemic control and inulin-type fructans (ITF) supplementation [9].

ITF, mainly composed of inulin, fructooligosaccharides (FOS) and galactooligosaccharides (GOS), is a class of linear fructans that is connected with β (2-1) bonds and is often defined as one kind of prebiotic [10, 11]. Many studies have provided evidence that ITF has many health benefits, such as improving immune function [12], lowering blood pressure [13], and improving blood lipids [14] if taken at a moderate dose. Although a growing body of human clinical trials, including randomized controlled trials (RCTs), support that ITF intake plays an important role in glycemic control, the results have remained controversial [15].

A previous meta-analysis conducted by Liu [14] studied ITF effects on blood lipid profiles and two glycemic indicators [fasting blood glucose (FBG) and fasting insulin (FINS)] in 2016, but no significant result was found for FBG concentration. With more trials performed in recent years, ITF has shown a more strongly linked ability to improve glycemic control and insulin resistance. Moreover, there have been no studies systematically evaluating the association between ITF supplementation and two important glycemic indicators, glycosylated hemoglobin (HbA1c) and homeostasis model assessment-insulin resistance (HOMA-IR), which were related to long-term glycemic regulation and insulin sensitivity, respectively.

Therefore, we conducted a meta-analysis of all relevant RCTs to systematically assess the effects of ITF supplementation on the four main glycemic indicators (FBG, FINS, HbA1c and HOMA-IR), aiming at providing an evidence-based medical strategy for prediabetes and T2DM management in the clinic practice.

## Materials and methods

### Literature search

A systematic search was performed according to the guidelines of the 2009 Preferred Reporting Items for Systematic Reviews and Meta-Analysis (PRISMA) statement in the online databases PubMed, EMBASE, and the Cochrane Library until March 6, 2019. The following terms were used to search for related publications in titles and abstracts: (oligosaccharide OR fructooligosaccharide OR oligofructose OR inulin) AND (glycosylated hemoglobin OR HbA1c OR glucose OR fasting plasma glucose OR insulin resistance OR glycemic OR “HOMA”). The synonyms of terms, MESH terms and the wild card term ‘‘*’’ were also used in the search. The type of study was defined as a “clinical trial”. The language was restricted to English. The search strategies of the online databases are shown in Additional file 1: Table S1. If necessary, manual retrieval was also conducted to obtain additional relevant articles. The protocol was registered at PROSPERO (Registration Number: CRD42018115875).

### Study selection

Studies were included according to the following criteria: (i) primary RCT with either a parallel or crossover design; (ii) investigation of the impact of ITF supplementation on plasma/serum glycemic indicators (FBG, HbA1c, FINS, or HOMA-IR); (iii) treatment duration longer than 7 days; and (iv) sufficient glycemic index information at baseline and at the end of follow-up, or the net change values in each group needed to be provided.

Studies were excluded according to the following criteria: (i) intervention group used other carbohydrates than ITF, such as arabino-xylan and β-glucan; (ii) no appropriate control group for assessing the effect of ITF supplementation; (iii) duplicate studies; (iv) observational study design; or (v) the article was a meeting abstract.

With the inclusion criteria and exclusion criteria decided in advance, we completed the screening step by step: two authors (HH and PX) conducted the preliminary screening of the searched studies based on their titles and abstracts; then, they reviewed the full text to assess eligibility criteria independently. Final eligibility was determined through agreement between the 2 reviewers, with any disagreement resolved in consultation with LW.

### Data extraction

HH and PX independently extracted and cross-checked the following information from the included studies: basic information about the research (first author’s name, year of publication, study region, underlying disease of the study population and eligibility, study design, sponsor, sample size, numbers of participants who completed the study, numbers of participants used for analysis); subjects’ characteristic information (age, sex, body mass index, baseline glycemic parameters, and antidiabetic medication use); data on the intervention and control groups (kinds of ITF and control, food carrier, daily dose, duration of intervention); and outcomes of biomarkers for glucose and insulin homeostasis (FBG, HbA1c, FINS, and HOMA-IR); adverse reactions and reasons for loss of follow-up were also collected. Notably, if the necessary original data were not given but were presented by a column graph, we extracted the data according to the graph.

### Quality assessment

The methodological quality and the risk of bias of the included trials were independently assessed by two authors (HH and PX) using the Cochrane criteria. The seven assessment items used for the assessment of each study were as follows: adequacy of random sequence generation, allocation concealment, blinding of participants and personnel, outcome assessment, addressing of dropouts (incomplete outcome data), selective outcome reporting, and other potential sources of bias. These seven criteria were rated as ‘low risk’, ‘unclear risk’ or ‘high risk’ depending on the characteristics of each criterion reported in the study.

### Quantitative data synthesis

The meta-analysis was conducted using RevMan software version 5.2 (Cochrane Collaboration, Oxford, UK). The effect sizes were expressed as the weighted mean difference (WMD) and 95% confidence interval (CI). The WMD was estimated by calculating the net change of the mean difference by subtracting the baseline value after treatment in the intervention and control groups. The standard deviation (SD) was calculated using the method described by Hozo et al. [16] and Simental-Mendía et al. [17]. If there were different reporting units for the indexes in the original studies, a unit conversion calculation was performed.

Considering the included studies mainly performed according to health status, we categorized the participants into four groups: healthy, prediabetes and T2DM, overweight and obesity, and others. A random-effects model (using the DerSimonian-Laird method) and the generic inverse variance method were used to compensate for the heterogeneity of the studies in terms of the different groups. Interstudy heterogeneity was assessed using the Cochran Q test and I^2^ index and was regarded as substantial if I^2^ > 50% and *P* value was low (< 0.10).

#### Nonlinear dose–response analysis

We tested the dose–response relationship between ITF supplementation and the glycemic indicators with the nonlinear robust error meta-regression (REMR) model, which is mainly based on the inverse variance-weighted least squares regression and cluster robust error variances for dealing with the synthesis of correlated dose–response data from different studies. A detailed theoretical rationale and Stata codes can be found in the methodological paper of Xu and Doi [18].

### Subgroup analyses

Subgroup analyses were performed to estimate the effect size of ITF supplementation on glycemic indicators in different subsets of studies categorized according to the presence of potential confounders. Apart from the aforementioned healthy status, the studies were also categorized according to sex (male versus female), type of ITF (inulin versus other kinds), method of ITF intake (in drinks versus other kinds), intervention-control design (an ITF versus non-ITF design versus a synbiotic versus probiotic design), study design (parallel versus crossover), country of study (Iran versus other countries), and sponsor referred (no versus yes).

### Publication bias and sensitivity analysis

The potential publication bias was explored by using visual inspection of funnel plot asymmetry with RevMan software and Egger’s weighted regression for quantitative assessment with Stata 12.0 software (College Station, Texas 77845 USA).

To evaluate the influence of each study on the overall effect size, a sensitivity analysis was conducted using the leave-one-out method, i.e., iteratively removing one study and repeating the analysis. To further test the robustness of the results, sensitivity analyses were also used for excluding studies of high heterogeneity that changed the pooled result more than 10%, and data were reanalyzed using a fix-effects model for I^2^ < 50%.

### GRADE certainty of the body of evidence

The overall certainty of evidence across the studies was graded according to the guidelines of the GRADE (Grading of Recommendations Assessment, Development, and Evaluation) Working Group [19]. The quality of evidence could be classified into four categories according to the corresponding evaluation criteria: high, moderate, low, and very low [20].

## Results

### Flow and characteristics of the included studies

**Fig. 1:**
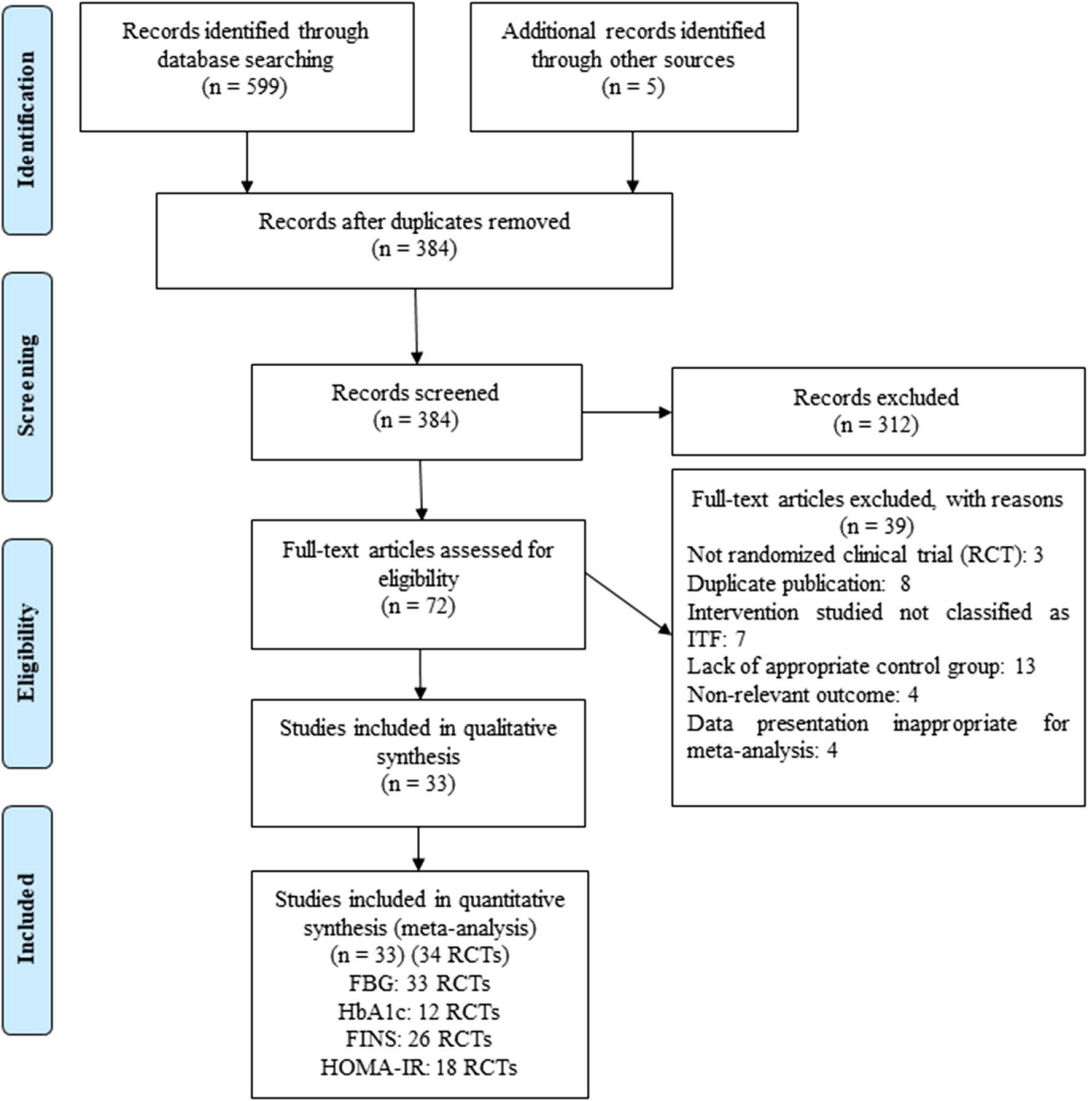
PRISMA flowchart indicating studies identified by and included in the systematic review

The detailed process of the PRISMA flowchart is presented in Fig. 1. Initially, 599 published studies were identified after searching multiple databases, and 5 additional records were identified through other sources. After carefully screening and assessing eligibility, 33 studies [21–53] were found eligible and were included in the systematic review. It was noteworthy that one trial [47] included two well-matched RCTs and actually counted as 2 RCTs in our meta-analysis. Therefore, 33 trials (34 RCTs) were included in all.

The study characteristics are presented in Table 1. Thirty-three clinical trials [21–53] involving 1346 participants were included, among which 23 trials were parallel RCTs [22–26, 28–35, 37, 39, 40, 44–47, 49–51] and 10 were crossover RCTs [21, 27, 36, 38, 41–43, 48, 52, 53]. Five studies [33, 39, 41, 46, 48] were conducted in healthy subjects, 7 studies [26, 28, 32, 44, 51–53] in the overweight and obesity populations and 14 studies (15 RCTs) [21, 22, 25, 29–31, 34, 35, 37, 38, 42, 45, 47, 49] in the prediabetes and T2DM population, and 7 studies [23, 24, 27, 36, 40, 43, 49] in the other group were performed mainly in non-alcoholic steatohepatitis patients [23, 24, 27, 40] or the elderly [43, 49]. The intervention substances varied among the included studies: 12 studies [25, 30, 34, 35, 37–40, 47, 48, 51, 52] used inulin only, and 4 studies [22, 23, 46, 50] used synbiotic (a combination of ITF and probiotics). The daily dose and duration of the intervention period varied between studies. The daily dose of ITF ranged from 5.5 to 30 g (median dose: 10 g/day), and the duration of the intervention periods ranged from 20 to 252 days (median duration: 56 days). Eligible outcomes of glycemic indicators were reported: FBG in 32 studies (33 RCTs) [21–37, 39–53], HbA1c in 11 studies (12 RCTs) [25, 29–31, 34, 35, 42, 45, 47], FINS in 25 studies (26 RCTs) [22–24, 26–30, 32–35, 37–42, 45–49, 52, 53], and HOMA-IR in 17 studies (18 RCTs) [22–24, 28, 30, 32, 34, 35, 37, 38, 40, 45–49, 52]. Except for one study [23], in which subjects were instructed to modify dietary intake in both the intervention and control groups, participants were advised to maintain their usual diet. The side effects were studied in 26 trials, and not mentioned in 7 others. Of the 26 trials, 19 explicitly reported all participants in the intervention and control groups had no adverse effects after substances supplementation, 5 showed no significant difference in the incidence of adverse effects between participants of the intervention and control groups, except some subjects in 2 studies [43, 44] were reported to suffer intestinal pressure, flatulence or abdominal discomfort.

**Table 1:** Characteristics of the included studies

References, country	Participants					Intervention		Comparison	Outcome^a^	Study design
	N (I/C)	Age	Gender	BMI	Population	Type	Dosage			
Alles [21], Netherlands	20 (20/20)	59	M-9 F-11	28.3 ± 3.5	T2DM	FOS	15 g/days (20 days)	Glucose	↔FBG	C, RCT, SB
Asemi [22], Iran	54 (27/27)	35–70	M and F	30.4 ± 5.3	T2DM	Inulin and *Lactobacillus sporogenes*	8.4 g/days (56 days)	*L. sporogenes* bread	↔FBG, ↓FINS, HbA1c	P, RCT, DB
Behrouz [23], Iran	59 (29/30)	20–60	M and F	25–40	Nonalcoholic fatty liver disease	Oligofructose and probiotic	16 g/days (84 days)	Maltodextrin	↔FBG, FINS, HbA1c	P, RCT, DB
Bomhof [24], Canada	14 (8/6)	≥ 18	M-8 F-6	> 25	non-alcoholic steatohepatitis with overweight	Oligofructose	16 g/days (252 days)	Maltodextrin	↔FINS, HOMA-IR, ↑FBG	P, RCT, DB
Bonsu [25], Canada	26 (12/14)	> 40	M and F	30.3 ± 4.4	T2DM	Inulin	10 g/days (84 days)	Xylitol	↔FBG, HOMA-IR	P, RCT, DB
Canfora [26], Netherlands	44 (21/23)	45–70	M and F	28–40	Overweight or obese	GOS	15 g/days (84 days)	Maltodextrin	↔FBG, FINS	P, RCT, DB
Daubioul [27], Italy	7 (7/7)	48–60	M and F	21.7–37.6	Non-alcoholic steatohepatitis	FOS	16 g/days (56 days)	Maltodextrin	↔FBG, FINS	C, RCT, DB
de Luis [28], Spain	32 (16/16)	25–60	M and F	30–35	Obese	FOS	9.84 g/days (30 days)	Control cookie	↔FBG, FINS, HbA1c	P, RCT, DB
Dehghan [31], Iran	52 (27/25)	20–65	F-only	25–34.99	T2DM	Oligofructose and inulin	10 g/days (56 days)	Maltodextrin	↓FBG, HOMA-IR	P, RCT, TB
Dehghan [32], Iran	49 (24/25)	20–65	F-only	> 25	T2DM	Inulin	10 g/days (56 days)	Maltodextrin	↓FBG, FINS, HbA1c, HOMA-IR	P, RCT, DB
Dehghan [29], Iran	49 (27/22)	30–65	F-only	25–34.99	T2DM	Oligofructose and inulin	10 g/days (60 days)	Maltodextrin	↓FBG, HOMA-IR, ↔ FINS	P, RCT, DB
Dewulf [32], Belgium	30 (15/15)	18–65	F-only	> 30	Obese	Inulin and oligofructose	16 g/days (90 days)	Maltodextrin	↔FBG, FINS, HbA1c, HOMA-IR	P, RCT, DB
Forcheron [33], France	17 (9/8)	31.7 ± 4.0	M-6 F-11	ND	Healthy	Inulin and oligofructose	10 g/days (180 days)	Maltodextrin	↔FBG, FINS	P, RCT, DB
Gargari [34], Iran	49 (24/25)	20–65	F-only	25–35	T2DM	Inulin	10 g/days (60 days)	Maltodextrin	↓FBG, FINS, HbA1c, HOMA-IR	P, RCT, TB
Ghavami [35], Iran	46 (23/23)	30–50	M and F	25–35	T2DM	Inulin	10 g/days (42 days)	Starch	↔FBG, FINS, HbA1c, ↓HOMA-IR	P, RCT, DB
Giacco [36], Italy	30 (30/30)	45.5 ± 9.9	M-20 F-10	26.6 ± 2.2	Mild hypercholesterolaemia	FOS	10.6 g/days (60 days)	Maltodextrine plus aspartame	↔FBG	C, RCT, DB
Guess [37], UK	38 (20/18)	> 18	M and F	25–35	Prediabetes	Inulin	30 g/days (126 days)	Cellulose	↓FBG, FINS, HbA1c	P, RCT, DB
Guess [38], UK	34 (34/34)	≥ 18	M and F	25–35	Overweight subjects with prediabetes	Inulin	30 g/days (42 days)	Cellulose	↔FINS, ↓HbA1c	C, RCT, DB
Jackson [39], UK	54 (27/27)	35–65	M and F	20–32	Healthy	Inulin	10 g/days (56 days)	Maltodextrin	↔FBG, FINS	P, RCT, DB
Javadi [40], Iran	38 (19/19)	20–60	M and F	30.7 ± 3.7	Non-alcoholic steatohepatitis	Inulin	10 g/days (90 days)	Maltodextrin	↔FBG, ↓FINS, HbA1c	P, RCT, DB
Luo [41], France	12 (12/12)	19–32	M-only	21.0 ± 1.7	Healthy	FOS	20 g/days (28 days)	Sucrose	↔FBG, FINS	C, RCT, DB
Luo [42], Belgium	10 (10/10)	57.0 ± 6.3	M-6 F-4	28.0 ± 3.2	T2DM	FOS	20 g/days (28 days)	Sucrose	↔FBG, FINS, HOMA-IR	C, RCT, DB
Meksawan [43], Thailand	9 (9/9)	> 50	M-5 F-4	ND	Elderly CAPD patients	FOS	20 g/days (30 days)	Sucrose	↔FBG	C, RCT, DB
Parnell [44], Canada	39 (21/18)	20–70	M and F	>25	Overweight or obese	Oligofructose	21 g/days (84 days)	Maltodextrin	↔FBG	P, RCT, DB
Pedersen [45], UK	29 (14/15)	42–65	M-only	28.2 ± 1.0	T2DM	GOS	5.5 g/days (84 days)	Maltodextrin	↔FBG, FINS, HbA1c, HOMA-IR	P, RCT, DB
Rajkumar [46], India	30 (15/15)	20–25	M-14 F-16	18.5–24.9	Healthy	FOS and *L. salivarius*	10 g/days (42 days)	*L. salivarius*	↔FBG, FINS, HbA1c	P, RCT, SB
Roshanravan [47], Iran	30 (15/15)	30–55	M and F	27–35	T2DM	Inulin	10 g/days (45 days)	Starch	↔FBG, FINS, HbA1c, HOMA-IR	P, RCT, DB
Russo [48], Italy	15 (15/15)	18.8 ± 0.7	M-only	22.8 ± 2.3	Healthy	Inulin	11 g/days (35 days)	Control pasta	↔FBG, HbA1c, HOMA-IR, ↑FINS	C, RCT, DB
Scheid [49], Brazil	72 (35/37)	67.1 ± 6.1	M and F	27.9 ± 5.0	Elderly	FOS	7.4 g/days (63 days)	Maltodextrin	↔FBG, FINS, HbA1c	P, RCT, DB
Shakeri [50], Iran	52 (26/26)	53	M and F	30.3 ± 5.3	T2DM	Inulin and *Lactobacillus sporogenes*	8.4 g/days (56 days)	Lactobacillus sporogenes control breads	↔FBG	P, RCT, DB
Tovar [51], Mexican	59 (30/29)	18–50	F-only	≥ 25	Obese	Inulin	10 g/days (90 days)	No treatment	↔FBG	P, RCT, DB
Tripkovic [52], UK	10 (10/10)	30–55	M-only	25–35	Overweight	Inulin	15 g/days (28 days)	Control bread rolls	↔FBG, FINS, HbA1c	C, RCT, DB
Vulevic [53], UK	45 (45/45)	18–65	M-16 F-29	> 25	Overweight	GOS	5.5 g/days (84 days)	Maltodextrin	↔FBG, ↓FINS	C, RCT, DB

*C* Cross-over, *CAPD* continuous ambulatory peritoneal dialysis, *DB* double-blinded, *FBG* fasting blood glucose, *FINS* fasting insulin, *FOS* fructooligosaccharide, *GOS* galactooligosaccharides, *HbA1c* glycosylated hemoglobin, *HOMA*-*IR* homeostasis model assessment-insulin resistance, *ND* no data, *P* Parallel, *RCT* randomized controlled trial, *SB* single-blinded, *TB* triple-blinded, *T2DM* Type 2 diabetes

^**a**^↑: means the glycemic indicator(s) in the ITF intervention group was (were) significantly increased while compared with their control group; ↔, means the glycemic indicator(s) in the ITF intervention group was (were) not significantly changed while compared with their control group; ↓, means the glycemic indicator(s) in the ITF intervention group was (were) significantly decreased while compared with their control group

### Study quality

The quality of bias assessment of the included studies is shown in Additional file 2: Figure S1. According to the seven assessment criteria of the Cochrane Handbook for Systematic Review of Interventions, most of the studies had good quality although some were characterized by insufficient information among the random sequence generation, allocation concealment, binding of outcome assessment and other bias, which was on account of the financial or food assistance provided by companies. In addition, bias may exist in some studies because 3 trials [25, 32, 48] had a high dropout rate.

### Main outcomes and GRADE certainty

We conducted a meta-analysis to assess the effect of ITF on glycemic indicators, including FBG, HbA1c, FINS, and HOMA-IR, and used GRADE to assess the results. The GRADE evidence profile for the summary of findings is presented in Table 2.

**Table 2:** GRADE profile of ITF supplementation for FBG, HbA1c, and FINS levels and HOMA-IR scores in the total population and in the prediabetes and T2DM population

Quality assessment						Summary of findings			Quality of evidence
Outcomes	Risk of bias	Inconsistency	Indirectness	Imprecision	Publication Bias	Number of intervention/control	Absolute effect (95% CI)	Relative effect^a^ intervention/control	
FBG (Total population)	No serious limitations	Serious limitations^b^	No serious limitations	No serious limitations	No serious limitations	656/651	− 0.21 (− 0.33, − 0.09) mmol/l	− 4.32%/0.36%	⊕ ⊕ ⊕○ moderate
HbA1C (Total population)	No serious limitations	Serious limitations^c^	No serious limitations	No serious limitations	No serious limitations	220/219	− 0.39 (− 0.65, − 0.13) %	− 4.93%/1.20%	⊕ ⊕ ⊕○ moderate
FINS (Total population)	No serious limitations	Very serious limitations^d^	No serious limitations	No serious limitations	Strongly suspected^e^	515/514	− 1.22 (− 1.90, − 0.54) μU/ml	− 12.62%/0.44%	⊕ ○○○ very low
HOMA-IR (Total population)	No serious limitations	Very serious imitations^f^	No serious limitations	No serious limitations	No serious limitations	357/360	− 0.57 (− 0.84, − 0.31)	− 19.10%/− 1.27%	⊕ ⊕ ○○ low
FBG (T2DM and prediabetes)	No serious limitations	No serious limitations	No serious limitations	No serious limitations	No serious limitations	283/280	− 0.60 (− 0.71, − 0.48) mmol/l	− 7.41%/− 0.26%	⊕ ⊕ ⊕ ⊕ high
HbA1C (T2DM and prediabetes)	No serious limitations	No serious limitations	No serious limitations	No serious limitations	No serious limitations	190/189	− 0.58 (− 0.83, − 0.32) %	− 5.15%/1.55%	⊕ ⊕ ⊕ ⊕ high
FINS (T2DM and prediabetes)	No serious limitations	Very serious limitations^g^	No serious limitations	No serious limitations	No serious limitations	232/229	− 1.75 (-2.87, − 0.63) μU/ml	− 20.08%/− 3.50%	⊕ ⊕ ○○ low
HOMA-IR (T2DM and prediabetes)	No serious limitations	Very serious limitations^h^	No serious limitations	No serious limitations	No serious limitations	195/197	− 0.69 (− 1.10, − 0.28)	− 27.63%/− 2.29%	⊕ ⊕ ○○ low

*FBG* fasting blood glucose, *FINS* fasting insulin, *HbA1c* glycosylated hemoglobin, *HOMA*-*IR* homeostasis model assessment- insulin resistance, *T2DM* type 2 diabetes mellitus

^a^The relative effect is computed by calculating the weighted mean of the baseline and hypoglycemic effects

^b^The test for heterogeneity is significant, and the I^2^ is moderate, 59%

^c^The test for heterogeneity is significant, and the I^2^ is moderate, 51%

^d^The test for heterogeneity is significant, and the I^2^ represent substantial heterogeneity, 69%

^e^The Egger’s test for publication bias is significant (*P *= 0.0035)

^f^The test for heterogeneity is significant, and the I^2^ represent substantial heterogeneity, 64%

^g^The test for heterogeneity is significant, and the I^2^ represent substantial heterogeneity, 78%

^h^The test for heterogeneity is significant, and the I^2^ represent substantial heterogeneity, 81%

**Fig. 2:**
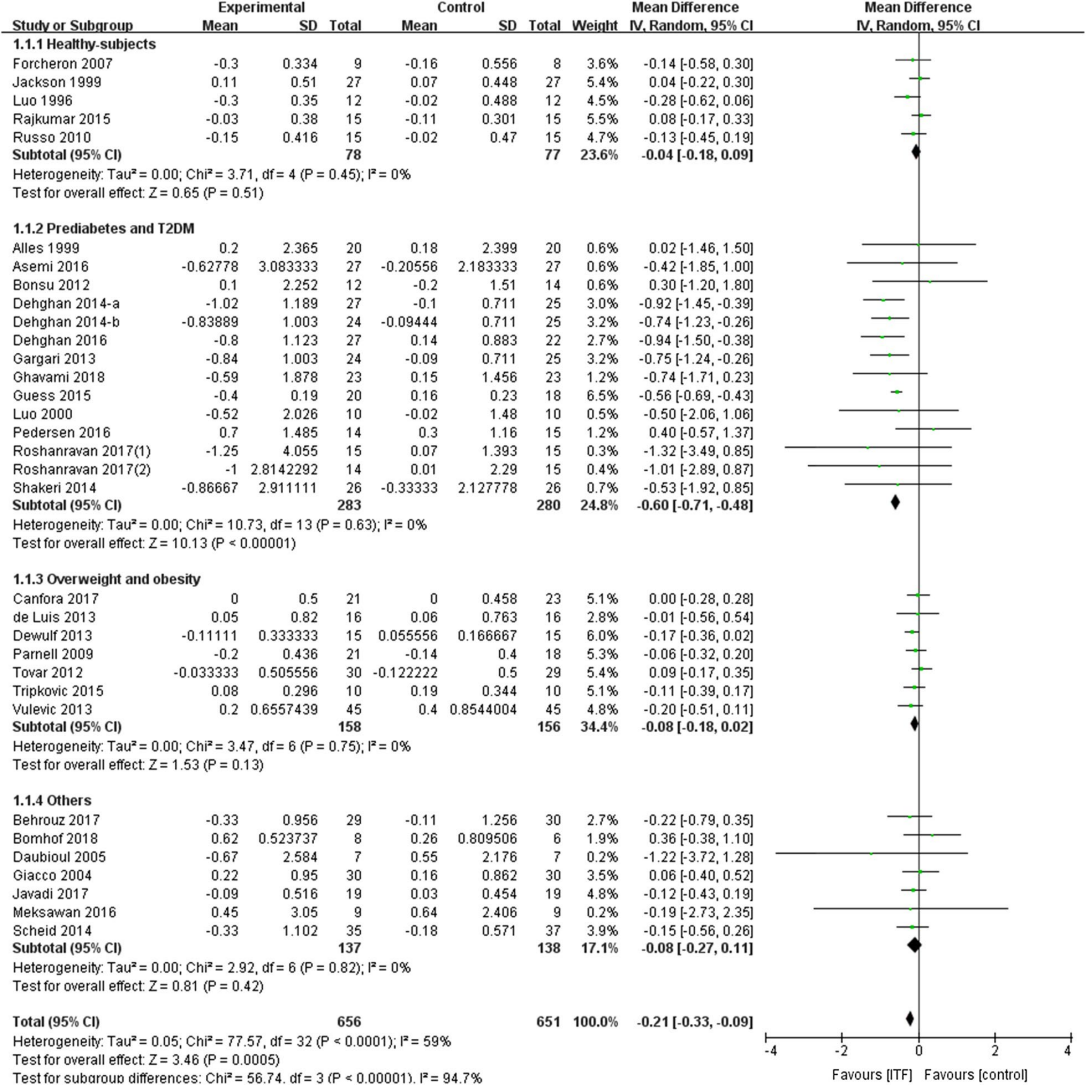
Forest plot displaying the effects of inulin-type fructans on fasting blood glucose (mmol/l) by subgroup

To explore whether ITF supplementation affected hyperglycemia, FBG data were analyzed. The effects of ITF on FBG were reported in 33 RCTs, including 14 RCTs in the prediabetes and T2DM population. The overall meta-analysis showed that ITF supplementation significantly reduced FBG with a WMD of − 0.21 mmol/l (95% CI − 0.33, − 0.09 mmol/l; *P *= 0.0005) (moderate rate). However, significant heterogeneity was observed between studies (I^2^ = 59%, *P *< 0.0001) (Fig. 2). Importantly, we found a more significant reduction in FBG based on the prediabetes and T2DM population (WMD: − 0.60 mmol/l; 95% CI − 0.71, − 0.48 mmol/l; *P *< 0.00001) (high rate), but the reduction was not significant in other populations. Moreover, no heterogeneity was observed in the grouped analyses with all *I*^2^ = 0%.

**Fig. 3:**
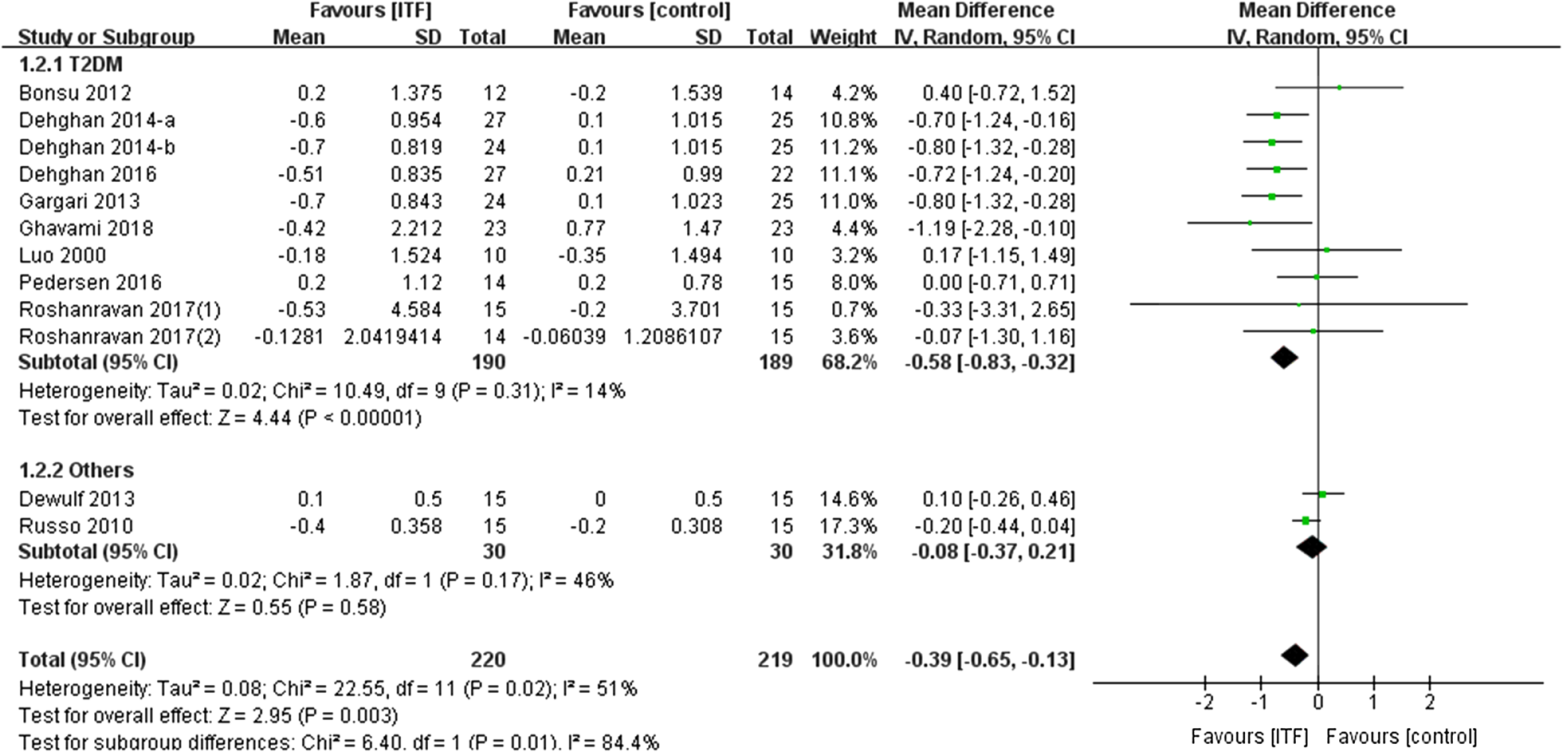
Forest plot displaying the effects of inulin-type fructans on glycosylated hemoglobin (%) by subgroup

Next, we examined whether ITF supplementation affected long-term glycemic regulation by analyzing HbA1c data. The effect of ITF on HbA1c was reported in 12 RCTs, including 10 RCTs in the prediabetes and T2DM population. The overall analysis revealed that HbA1c was reduced significantly (WMD: − 0.39%; 95% CI − 0.65, − 0.13%; *P *= 0.003) (moderate rate). Notably, in the T2DM population, HbA1c showed a significant reduction with a WMD of − 0.58% (95% CI − 0.83, − 0.32%; *P *< 0.00001) (high rate), and no significant heterogeneity was observed across studies (I^2^ = 14%, *P *= 0.31) (Fig. 3).

**Fig. 4:**
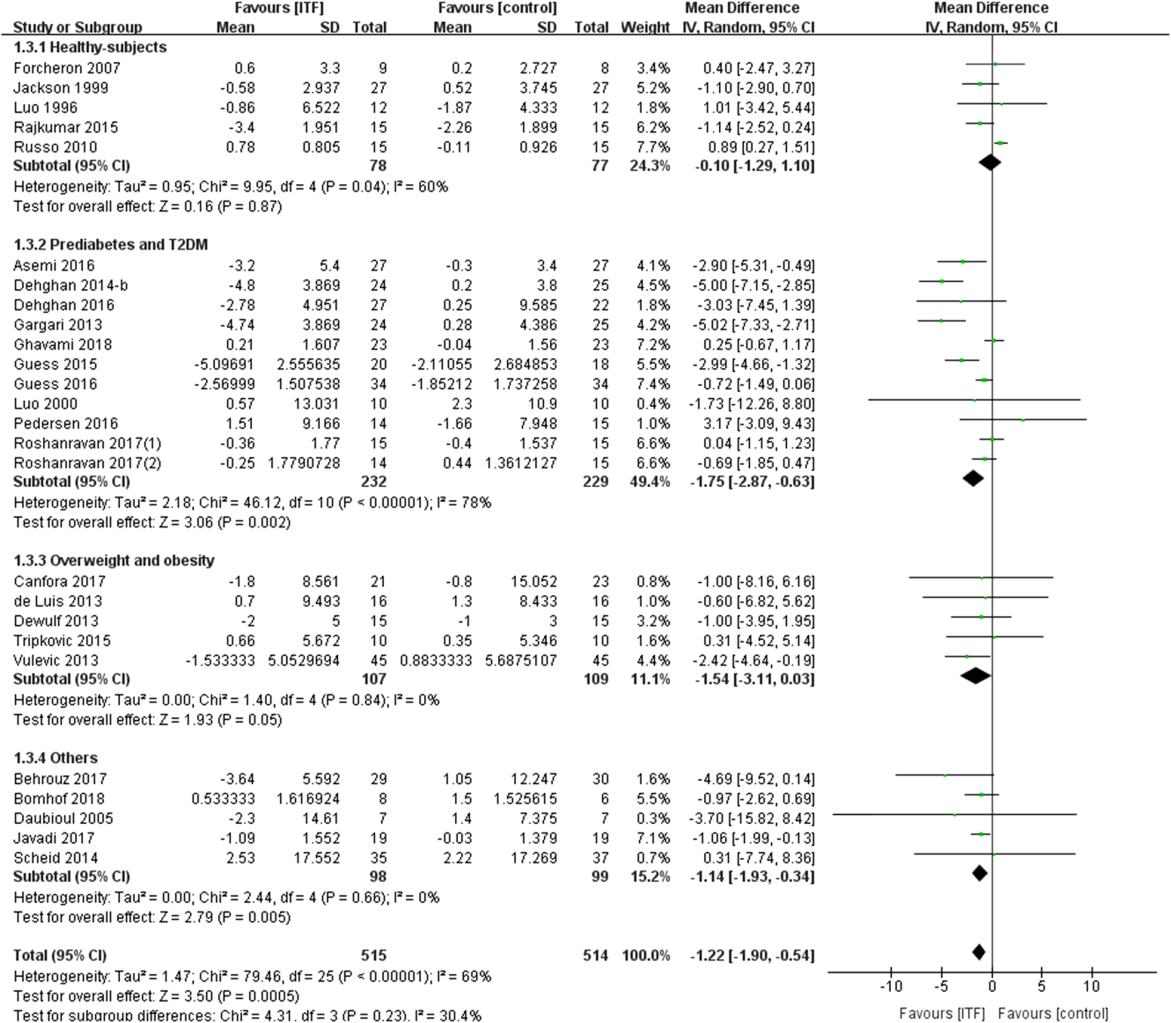
Forest plot displaying the effects of inulin-type fructans on fasting insulin (μU/ml) by subgroup

Next, we analyzed the effect of ITF on the fasting insulin concentration. Twenty-six RCTs reported changes in FINS after ITF supplementation, including 11 RCTs in the prediabetes and T2DM population. Overall, ITF supplementation reduced FINS significantly with a WMD of − 1.22 µU/ml (95% CI − 1.90, − 0.54 µU/ml; *P *= 0.0005) (very low rate) (Fig. 4). In the prediabetes and T2DM population, FINS showed a more significant reduction (WMD: − 1.75 µU/ml; 95% CI − 2.87, − 0.63 µU/ml; *P *= 0.002) (low rate), and the reduction was not significant or was modestly significant in the other populations.

**Fig. 5:**
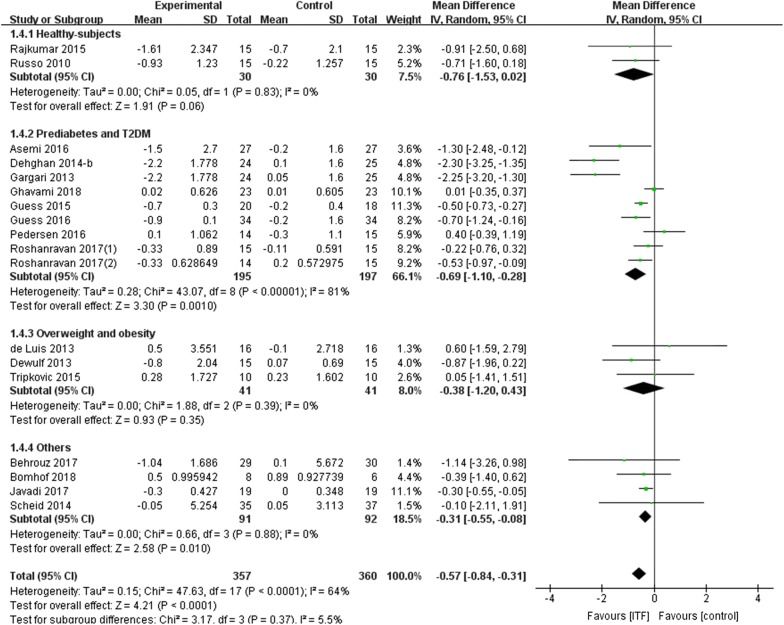
Forest plot displaying the effects of inulin-type fructans on homeostasis model assessment-insulin resistance (arbitrary units) by subgroup

Last, we also examined whether ITF supplementation affected insulin sensitivity by analyzing HOMA-IR. The effect of ITF on HOMA-IR was reported in 18 RCTs, among which 9 RCTs were based on the prediabetes and T2DM population. The overall analysis revealed that ITF supplementation significantly reduced HOMA-IR with a WMD of -0.57 (95% CI − 0.84, − 0.31; *P *< 0.0001) (low rate) (Fig. 5). In the prediabetes or T2DM subgroup, HOMA-IR also showed a significant reduction (WMD: − 0.69, 95% CI − 1.10, − 0.28, *P *= 0.001; I^2^ = 81%) (low rate).

In addition, to analyze the relative hypoglycemic effects, we calculated the weighted mean of the baseline and the hypoglycemic results of the four glycemic indicators (FBG, HbA1c, FINS, and HOMA-IR). In the prediabetes and T2DM population, compared with their control groups, the relative reduction of the four indicators reached − 7.15%, − 7.00%, − 16.58%, and − 25.34% of their baseline values in the supplementation group, which were − 4.68%, − 6.13%, − 13.06%, and − 17.83% in the total population, respectively. The relative effects in both the intervention and control groups are shown in Table 2.

### Nonlinear dose–response analysis

**Fig. 6:**
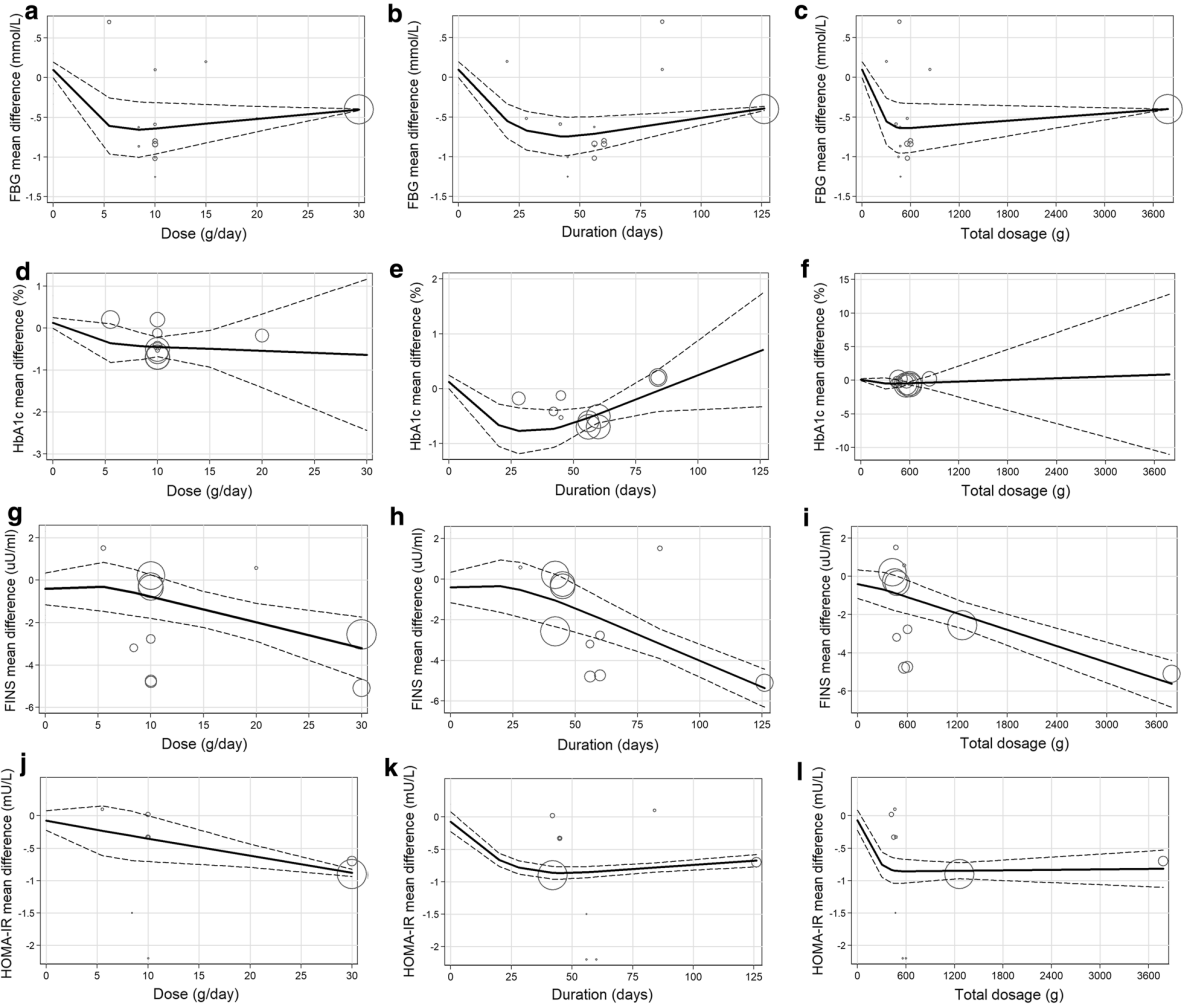
**a** analysis between dose of inulin-type fructans and FBG net change level; **b** analysis between duration of inulin-type fructans and FBG net change level; **c** analysis between total dosage of inulin-type fructans and FBG net change level; **d** analysis between dose of inulin-type fructans and HbA1c net change level; **e** analysis between duration of inulin-type fructans and HbA1c net change level; **f** analysis between total dosage of inulin-type fructans and HbA1c net change level; **g** analysis between dose of inulin-type fructans and FINS net change level; **h** analysis between duration of inulin-type fructans and FINS net change level; **i** analysis between total dosage of inulin-type fructans and FINS net change level; **j** analysis between dose of inulin-type fructans and HOMA-IR net change level; **k** analysis between duration of inulin-type fructans and HOMA-IR net change level; **l** analysis between total dosage of inulintype fructans and HOMA-IR net change level.

We explored the recommended daily dose and duration of ITF for glycemic control by dose–response analysis. As shown in Fig. 6, in the prediabetes and T2DM population, the relationship curves suggested that ITF supplementation had effects on the glycemic indicators, and the effects were different with different daily doses, durations and total doses of ITF. When the daily dose was 10 g and the duration reached 42 days and longer, these four glycemic indicators were significantly reduced, and the effect of glycemic control was satisfactory; the results were robust because the number of supporting studies was relatively large. Figure 6g, j, although FINS and HOMA-IR kept a decreasing trend when the daily dose was above 10 g, the supporting studies were fewer, and the results were not as credible. In the duration relationship curve, a similar situation existed.

These analyses were also performed in the total population (Additional file 3: Figure S2). The figure suggested that the overall trends of the curves were consistent with those of the prediabetes and T2DM population. For the HbA1c indicator, the trend of the dose–response relationship curves decreased rapidly at first and then rose gradually at some point.

### Subgroup analyses

The subgroup analysis results are presented in Table 3. The results showed that the female subgroup had reductions in FBG, HbA1c, FINS and HOMA-IR, while only FINS was significantly reduced in the male subgroup. The subgroup results showed that inulin had better effects on HbA1c and HOMA-IR than other kinds of ITF and that ITF supplementation in drinks had better effects on the four glycemic indicators than that in other foods, such as cookies, bread and so on. The pooled results of 4 studies examining symbiotic (ITF and probiotic) supplementation showed a significant reduction in FINS and HOMA-IR but no significant effects on the other two indicators. The study design, study country, and whether the mentioned sponsor might also be factors influencing the differences in the results between the studies.

**Table 3:** Subgroup meta-analysis of the effects of inulin-type fructans on glycemic indicators (FBG, HbA1c, FINS and HOMA-IR)

Subgroups	Fasting blood glucose (mmol/l)					HbA1c (%)					FINS (μU/ml)					HOMA-IR				
	n	WMD (95% CI)	*P*^a^	I^2^ (%)	*P*^b^	n	WMD (95% CI)	*P*^a^	I^2^ (%)	*P*^b^	n	WMD (95% CI)	*P*^a^	I^2^ (%)	*P*^b^	n	WMD (95% CI)	*P*^a^	I^2^ (%)	*P*^b^
Sex																				
Male	4	− 0.15 (− 0.32, 0.03)	0.106	0.0	0.599	2	− 0.18 (− 0.41, 0.05)	0.120	0.0	0.599	4	0.90 (0.30, 1.51)	0.004	71.2	< 0.001	3	− 0.09 (− 0.83, 0.66)	0.819	40.9	0.184
Female	6	− 0.51 (− 0.87, − 0.16)	0.004	81.7	< 0.001	5	− 0.56 (− 0.97, − 0.15)	0.008	72.3	0.006	4	− 0.37 (− 5.70, − 1.86)	< 0.001	47.7	0.125	3	− 1.84 (− 2.72, − 0.97)	< 0.001	56.9	0.098
ITF type																				
Inulin	12	− 0.28 (− 0.51, − 0.06)	0.012	74.5	< 0.001	7	− 0.48 (− 0.85, − 0.12)	0.010	47.6	0.076	11	− 1.20 (− 2.14, − 0.27)	0.012	84.5	< 0.001	10	− 0.63 (− 0.94, − 0.31)	< 0.001	76.9	< 0.001
Other kinds	21	− 0.15 (− 0.27, − 0.03)	0.011	25.3	0.142	5	− 0.28 (− 0.71, 0.14)	0.193	61.5	0.035	15	− 1.32 (− 2.07, − 0.56)	0.001	0.0	0.754	8	− 0.42 (− 0.92, 0.09)	0.108	18.9	0.281
Food based																				
ITF in drink	25	− 0.24 (− 0.39, − 0.09)	0.002	66.9	< 0.001	11	− 0.42 (− 0.74, − 0.11)	0.008	52.2	0.022	20	− 1.38 (− 2.06, − 0.70)	< 0.001	61.1	< 0.001	14	− 0.58 (− 0.87, − 0.28)	< 0.001	70.4	< 0.001
Others	8	− 0.12 (− 0.26, 0.03)	0.114	0.0	0.945	1	− 0.20 (− 0.44, 0.04)	0.101	~	~	6	− 0.28 (− 2.08, 1.51)	0.757	46.4	0.097	4	− 0.62 (− 1.27, 0.04)	0.067	9.2	0.347
Intervention type																				
ITF vs non-ITF	29	− 0.22 (− 0.35, − 0.10)	0.001	60.5	< 0.001	12	− 0.39 (− 0.65, − 0.13)	0.004	51.2	0.020	23	− 1.08 (− 1.81, − 0.36)	0.003	69.4	< 0.001	15	− 0.53 (− 0.81, − 0.25)	< 0.001	68.7	< 0.001
Synbiotics vs. probiotic	4	0.01 (− 0.21, 0.23)	0.944	0.0	0.697						3	− 2.10 (− 3.80, − 0.39)	0.016	34.5	0.217	3	− 1.16 (− 2.02, − 0.29)	0.009	0.0	0.928
Study design																				
Parallel	24	− 0.23 (− 0.39, − 0.08)	0.003	68.9	< 0.001	10	− 0.45 (− 0.77, − 0.13)	0.006	55.4	0.017	19	− 1.48 (− 2.25, − 0.71)	< 0.001	63.2	< 0.001	15	− 0.58 (− 0.89, − 0.28)	< 0.001	69.5	< 0.001
Cross-over	9	− 0.15 (− 0.30, − 0.01)	0.038	0.0	0.962	2	− 0.19 (− 0.42, 0.05)	0.117	0.0	0.590	7	− 0.32 (− 1.57, 0.92)	0.612	62.7	0.013	3	− 0.63 (− 1.07, − 0.19)	0.005	0.0	0.629
Country of study																				
Iran	11	− 0.59 (− 0.83, − 0.35)	< 0.001	31.1	0.151	7	− 0.75 (− 1.00, − 0.50)	< 0.001	0.0	0.919	9	− 1.95 (− 3.18, − 0.73)	0.002	80.4	< 0.001	8	− 0.82 (− 1.30, − 0.34)	0.001	82.1	< 0.001
Other countries	22	− 0.10 (− 0.22, 0.02)	0.116	58.8	< 0.001	5	− 0.08 (− 0.27, 0.11)	0.399	0.0	0.586	17	− 0.76 (− 1.56, 0.04)	0.062	52.8	0.006	10	− 0.48 (− 0.66, − 0.29)	< 0.001	0.0	0.523
Sponsor referred																				
No	25	− 0.27 (− 0.41, − 0.13)	< 0.001	60.0	< 0.001	11	− 0.42 (− 0.70, − 0.15)	0.003	54.0	0.017	20	− 1.32 (− 2.10, − 0.55)	0.001	74.0	< 0.001	13	− 0.63 (− 0.92, − 0.34)	< 0.001	70.2	< 0.001
Yes	8	− 0.01 (− 0.14, 0.13)	0.920	0.0	0.791	1	0.00 (− 0.71, 0.71)	1.000	~	~	6	− 1.01 (− 2.19, 0.17)	0.093	7.9	0.366	5	− 0.30 (− 1.04, 0.44)	0.422	37.8	0.169

*FBG* fasting blood glucose, *FINS* fasting insulin, *HbA1c* glycosylated hemoglobin, *HOMA*-*IR* homeostasis model assessment-insulin resistance, *ITF* inulin-type fructans, *WMD* weighted mean difference

^a^*P* value for subgroup differences between groups

^b^*P* value for heterogeneity within each subgroup

### Sensitivity analysis

The results of the leave-one-out sensitivity analysis suggested that the effects of ITF supplementation on all four glycemic indicators were robust and not significantly driven by any single study (Additional file 4: Figure S3).

In further sensitivity analyses, after the removal of high potential outlier studies that shifted the pooled mean difference more than 10%, the reanalysis results from the fixed effect model revealed no significant change after the exclusion compared with before. All the reanalysis results are summarized in Table 4.

**Table 4:** Reanalysis for the effects of inulin-type fructans on glycemic indicators (FBG, HbA1c, FINS and HOMA-IR) excluding high heterogeneity studies

Subgroups^a^	FBG (mmol/l)					HbA1c (%)					FINS (μU/ml)					HOMA-IR				
	n	WMD (95% CI)	*P*^b^	I^2^ (%)	*P*^c^	n	WMD (95% CI)	*P*^b^	I^2^ (%)	*P*^c^	n	WMD (95% CI)	*P*^b^	I^2^ (%)	*P*^c^	n	WMD (95% CI)	*P*^b^	I^2^ (%)	*P*^c^
Healthy-subjects	5	− 0.04 (− 0.18, 0.09)	0.513	0.0	0.447						5	− 0.10 (− 1.29, 1.10)	0.874	59.8	0.041	2	− 0.76 (− 1.53, 0.02)	0.056	0.0	0.830
T2DM and prediabetes	11	− 0.58 (− 0.86, − 0.31)	< 0.001	0.0	0.672	7	− 0.37 (− 0.71, − 0.02)	0.036	18.7	0.287	8	− 1.14 (− 2.05, − 0.23)	0.014	49.7	0.052	6	− 0.47 (− 0.64, − 0.30)	< 0.001	39.3	0.143
Overweight and obesity	7	− 0.08 (− 0.18, 0.02)	0.126	0.0	0.748						5	− 1.54 (− 3.11, 0.03)	0.054	0.0	0.844	3	− 0.38 (− 1.20, 0.43)	0.353	0.0	0.391
Others	7	− 0.08 (− 0.18, 0.02)	0.419	0.0	0.748	1	− 0.20 (− 0.44, 0.04)	0.101	~	~	5	− 1.14 (− 1.93, − 0.34)	0.005	0.0	0.655	4	− 0.31 (− 0.55, − 0.08)	0.010	0.0	0.883
Total	30	− 0.10 (− 0.18, − 0.03)	0.005	4.4	0.397	8	− 0.25 (− 0.45, − 0.06)	0.011	12.5	0.333	23	− 0.89 (− 1.50, − 0.28)	0.004	51.6	0.002	15	− 0.43 (− 0.56, − 0.29)	< 0.001	0.0	0.553

*FBG* fasting blood glucose, *FINS* fasting insulin, *HbA1c* glycosylated hemoglobin, *HOMA*-*IR* homeostasis model assessment- insulin resistance, *T2DM* type 2 diabetes mellitus

^a^Excluded trials: FBG: Dehghan [30], Dehghan [29], and Guess [37]; HbA1c: Dehghan [31], Dehghan [29], Gargari [34] and Dewulf [32]; FINS: Dehghan [31], Gargari [34] and Ghavami [35]; HOMA-IR: Dehghan [31], Gargari [34] and Ghavami [35]

^b^*P* value for subgroup differences between groups

^c^*P* value for heterogeneity within each subgroup

### Publication bias analyses

The publication bias of the included studies on the four indicators was inspected with a funnel plot and Egger’s test, and the results are shown in Additional file 5: Figure S4. The funnel plots of FBG, HbA1c and HOMA-IR were symmetrical, which may be interpreted as no publication bias and the same results were shown in Egger’s test (*P *> 0.05). However, the funnel plot and Egger’s test showed that there might be publication bias in the FINS results (t = − 2.24; 95% CI − 2.28, − 0.09; *P* = 0.035).

## Discussion

In this systematic review and meta-analysis of 33 RCTs involving a total of 1346 participants, we assessed the effects of ITF supplementation on four glycemic indicators, including FBG, HbA1c, FINS and HOMA-IR scores. In this regard, this meta-analysis provides the most up-to-date evidence supporting the putative favorable effects of ITF supplementation on glycemic control. Indeed, the results of our study showed that ITF supplementation could modulate glycemic control in the total population, and better effects were found in the prediabetes and T2DM population. The REMR results revealed that when supplementing ITF with a daily dosage of 10 g and a duration of 6 weeks and longer, the glycemic indicators of the prediabetes and T2DM population were well controlled and that ITF supplementation was suitable for the total population with only modest albeit significant effects. In addition, the subgroup results showed that the sex of the subjects and the type and the method of intake of ITF were all important factors influencing the hypoglycemic effect of ITF.

Soluble dietary fiber, one kind of nondigestible carbohydrate, has been widely considered to play an important role in glycemic control, and recently, two meta-analyses (Thompson [54] and Silva [55]) both confirmed its effect on improving glycemic control. ITF, a common but important soluble dietary fiber, has also received much attention. In recent years, interest in the effects of ITF on glycemic control has increased considerably. From the current research results, the hypoglycemic effect of ITF may have several mechanisms. ITF, which is fermented in the intestine, delays the rate of gastric emptying, thereby slowing the flow of glucose into the bloodstream and reducing the extent of postprandial blood glucose elevation [56]. At the same time, the short-chain fatty acids of the fermentation products after ingestion, especially propionic acid, may reduce or inhibit hepatic gluconeogenesis. On the other hand, propionic acid enhances glucose utilization by consuming liver citric acid. Propionic acid may also indirectly affect hepatic glucose metabolism by reducing the concentration of plasma fatty acids, a known factor closely related to gluconeogenesis [57]. In addition, studies have shown that oligofructose can improve blood glucose metabolism by increasing the levels of glucagon-like peptide (GLP-1) and glucagon-like peptide 2 (GLP-2) [58, 59]. Jafarnejad et al. [60] reported that ITF, a type of prebiotic, could significantly reduce blood glucose by promoting probiotic regulatory immune responses and systemic lowering of inflammation.

The side effects are important to mention. In the included RCTs, 26 of which studied the side effects in both intervention and control groups, and seven did not mention. Among the 26 trials, most of which reported that ITF were well tolerated by all subjects, and only two studies [43, 44] reported that ITF were associated with minor side effects, such as slight abdominal flatulence or bloating, which may be important functional expressions of prebiotics as they are the result of gas and acid produced by fermentation by gut microbiota in the colon. None of these side effects were considered serious or harmful to health, and the side effects subsided with adaptation over time. Recently, one study [61] revealed that inulin supplementation was associated with liver damage and might even lead to liver cancer. However, in our included trials, even adverse effects on liver function were not reported. The reason might be that the subjects in these studies were mice, a different species from humans. In addition, inulin dosage might also be an important factor to consider. The China Ministry of Health Announcement No. 5 of 2009 approved inulin and polyfructose as new resource foods and stated that the recommended consumption was less than 15 g per day. The Generally Recognized as Safe Notice (GRN) No. 605 mentioned that, in the general population, exposure to FOS in food at levels up to 20 g/day was considered safe. In addition, the taste of inulin was generally accepted, and the price was relatively low, which might make it a possible substitute for sugar in the diet.

In 2017, one meta-analysis conducted by Liu [14] studied the effects of ITF on blood lipid and blood sugar levels. Their meta-analysis mainly focused on the effect of ITF in a narrow population of individuals with dyslipidemia, which resulted in a limited number of trials and a small sample of glycemic control studies. Their results showed that ITF supplementation significantly reduced blood lipid parameters. However, no significant reduction in FBG was identified in the T2DM population (MD: − 0.42 mmol/l; 95% CI − 0.90, 0.06 mmol/l; *P* = 0.09), with only three RCTs included in the T2DM subgroup analysis. In addition, HbA1c and HOMA-IR, both of which are quite important in glycemic control, were not included in their meta-analysis. Recently, more and more relevant well-designed RCTs have been reported, allowing us to perform a more specific and comprehensive meta-analysis to investigate the effects of ITF supplementation on glycemic control.

Our updated meta-analysis included 33 RCTs that evaluated the effect of ITF supplementation in all kinds of populations, especially prediabetes or T2DM subjects. First, the pooled results of our meta-analysis showed that ITF supplementation could improve glycemic control in the total population or in the prediabetes and T2DM population and that the results had good quality and recommendation levels after being assessed by GRADE. Moreover, except for FBG and FINS, we analyzed two other important glycemic indicators, HbA1c and HOMA-IR. HbA1c can reflect long-term glycemic control in diabetic patients, and a large number of studies have shown that a high level of HbA1c is a risk factor for diabetic complications [62, 63]. Importantly, the WMD calculated by net change was used in our study, which balanced the baseline difference among studies, making our results more accurate. Second, we analyzed and looked for causes of heterogeneity and appropriately conducted a subgroup analysis, which effectively reduced the effect of heterogeneity on the results. Notably, the effects of ITF were much stronger on glycemic control in the prediabetes and T2DM population. For example, the FBG concentration in the diabetic subgroup was significantly reduced by − 0.72 mmol/l, which was 6 to 7 times greater than the reduction in the total population (− 0.11 mmol/l). Finally, and notably, we performed a dose–response meta-analysis to provide specific suggestions for ITF intake for the prediabetes and T2DM population.

Several limitations of the present study deserve to be mentioned. (i) Some included studies had a small sample size, which may make them likely to report extremely large beneficial effects and have low methodological quality. However, a sensitivity analysis was conducted to exclude the outlier effects studies, and the remainder of the reanalysis results did not show any significant change with the omission of the studies from our meta-analysis. Moreover, the quality of the small trials was also critically analyzed, and only the high-quality studies were included in our analysis. (ii) Medication usage in the included studies may not have been the same, and more medical usage information could not be obtained, which may have caused bias in this meta-analysis, although ITF supplementation does not influence medication use. We performed a subgroup analysis, and no heterogeneity was found, with an I^2^ of 0%. (iii) Some studies did not meet the inclusion criteria because they did not report baseline characteristics (WMD could not be computed) and were not in English, which may improve the publication bias; fortunately, only the HbA1c index showed a slight bias. (iv) Though some articles have shown that glucose iAUC levels were also reduced after ITF supplementation, this blood glucose indicator was not analyzed in this study due to differences in implementation criteria and the relatively small numbers of studies. Our team will continue to focus on the impact of ITF on this indicator and conduct another meta-analysis after more high-quality studies are reported. Despite some shortcomings, this study was the most extensive meta-analysis evaluating the effects of ITF on glycemic metabolism.

## Conclusions

Our comprehensive meta-analysis indicated that ITF supplementation reduced the four main glycemic indicators significantly, thus improving glycemic control, especially for the prediabetes and T2DM population. Importantly, we first conducted a dose–response meta-analysis, and recommended an ITF supplementation of 10 g per day for 6 weeks and longer for the prediabetes and T2DM population. In addition, our subgroup analyses revealed that there were more beneficial effects on the glycemic indicators in subjects who were females, in subjects who took inulin (one type of ITF) and in subjects who took ITF as a drink. Therefore, all these important findings provide practical information and indicate that ITF can be used as an adjuvant therapy for glycemic control, especially for the patients with prediabetes or T2DM in clinical practice.

## Supplementary information

**Additional file 1: Table S1.** Search strategies in the online databases.

**Additional file 2: Figure S1.** Risk of bias graph (A) and risk of bias summary (B) in 33randomized controlled trials.

**Additional file 3: Figure S2.** Non-linear dose-response analysis between ITF supplement (dose, duration, and total dosage) and glycemic parameters (FBG, HbA1c, FINS, HOMA-IR) levels in the total population. The dose-response analysis was conducted using the nonlinear robust error meta-regression (REMR) model, which is mainly based on the inverse variance-weighted least squares regression and cluster robust error variances for dealing with the synthesis of correlated dose-response data from different studies. The solid line represents weighted mean difference and the dotted lines represent the 95% confidence intervals (CIs). Abbreviations: FBG, fasting blood glucose; FINS, fasting insulin; HbA1c, glycosylated hemoglobin; HOMA-IR, homeostasis model assessment-insulin resistance; T2DM, type2 diabetes mellitus.

**Additional file 4: Figure S3.** Sensitivity analysis of the included studies of FBG (A), HbA1c (B), FINS (C), and HOMA-IR (D).FBG, fasting blood glucose; FINS, fasting insulin; HbA1c, glycosylated hemoglobin; HOMA-IR, homeostasis model assessment-insulin resistance.

**Additional file 5: Figure S4.** Funnel plots for meta-analysis of inulin-type fructans onFBG (A), HbA1c (B), FINS (C), and HOMA-IR (D). FBG, fasting blood glucose; FINS, fasting insulin; HbA1c, glycosylated hemoglobin; HOMA-IR, homeostasis model assessment-insulin resistance.

## Data Availability

Not applicable.

## Abbreviations

CI: confidence interval
FBG: fasting blood glucose
FINS: fasting insulin
HbA1c: glycosylated hemoglobin
HOMA-IR: homeostasis model assessment-insulin resistance
ITF: inulin-type fructans
PRISMA: Preferred Reporting Items for Systematic Reviews and Meta-Analysis
RCTs: randomized controlled trials
T2DM: type 2 diabetes
WMD: weighted mean difference
