# Supplementary material for: Inulin-type fructans supplementation improves glycemic control for the prediabetes and type 2 diabetes populations: results from a GRADE-assessed systematic review and dose–response meta-analysis of 33 randomized controlled trials
Source: J Transl Med. 2019 Dec 5;17:410. doi: 10.1186/s12967-019-02159-0 (PMC6896694; doi:10.1186/s12967-019-02159-0)
Supplement: Supplementary file 1 — Additional file 1: Table S1. Search strategies in the online databases. [file 12967_2019_2159_MOESM1_ESM.pdf]

Additional file 1: Table S1. Search strategies in the online databases

Online databases	Search strategy	Number of records
Pubmed	((((((glycosylated hemoglobin[Title/Abstract] OR HBA1c[Title/Abstract]) OR HBALC[Title/Abstract]) OR glucose[Title/Abstract]) OR FPG[Transliterated Title]) OR Insulin resistance[Title/Abstract]) OR glycemic[Title/Abstract]) AND (((((fructo-oligosaccharide[Title/Abstract] OR fructooligosaccharide[Title/Abstract]) OR Inulin[Title/Abstract]) OR oligofructose[Title/Abstract]) OR fructan[Title/Abstract]) OR (oligosaccharide[Title/Abstract] OR oligosaccharide's[Title/Abstract] OR oligosaccharidealditols[Title/Abstract] OR oligosaccharideglycans[Title/Abstract] OR oligosaccharides[Title/Abstract] OR oligosaccharides'[Title/Abstract] OR oligosaccharides1[Title/Abstract] OR oligosaccharidesas[Title/Abstract])) AND Clinical Trial[ptyp]	140
EMBASE	('glycosylated hemoglobin':ab,ti OR 'HBA1c':ab,ti OR 'HBALC':ab,ti OR 'glucose':ab,ti OR 'FPG':ab,ti OR 'Insulin resistance':ab,ti OR 'Glycemic':ab,ti) AND ('fructo-oligosaccharide':ab,ti OR 'fructooligosaccharide':ab,ti OR 'inulin':ab,ti OR 'oligofructose':ab,ti OR 'fructan':ab,ti OR 'oligosaccharide':ab,ti OR 'oligosaccharides':ab,ti OR 'oligosaccharidealditols':ab,ti OR 'oligosaccharideglycans':ab,ti OR 'oligosaccharides':ab,ti OR 'oligosaccharides':ab,ti OR 'oligosaccharides1':ab,ti OR 'oligosaccharidesas':ab,ti) AND [controlled clinical trial]/lim	156
Cochrane	((glycosylated hemoglobin):ti,ab,kw OR (HBA1c):ti,ab,kw OR (HBALC):ti,ab,kw OR (glucose):ti,ab,kw OR (FPG):ti,ab,kw OR (Insulin resistance):ti,ab,kw OR (Glycemic):ti,ab,kw) AND ((fructo-oligosaccharide):ti,ab,kw OR (fructooligosaccharide):ti,ab,kw OR (inulin):ti,ab,kw OR (oligofructose):ti,ab,kw OR (fructan):ti,ab,kw OR (oligosaccharide):ti,ab,kw OR (oligosaccharides):ti,ab,kw OR (oligosaccharidealditols):ti,ab,kw OR (oligosaccharideglycans):ti,ab,kw OR (oligosaccharides):ti,ab,kw OR (oligosaccharides):ti,ab,kw OR (oligosaccharides1):ti,ab,kw OR (oligosaccharidesas):ti,ab,kw)	303
