# Supplementary material for: Inulin-type fructans supplementation improves glycemic control for the prediabetes and type 2 diabetes populations: results from a GRADE-assessed systematic review and dose–response meta-analysis of 33 randomized controlled trials
Source: J Transl Med. 2019 Dec 5;17:410. doi: 10.1186/s12967-019-02159-0 (PMC6896694; doi:10.1186/s12967-019-02159-0)
Supplement: Supplementary file 2 — Additional file 2: Figure S1. Risk of bias graph (A) and risk of bias summary (B) in 33randomized controlled trials. [file 12967_2019_2159_MOESM2_ESM.pdf]

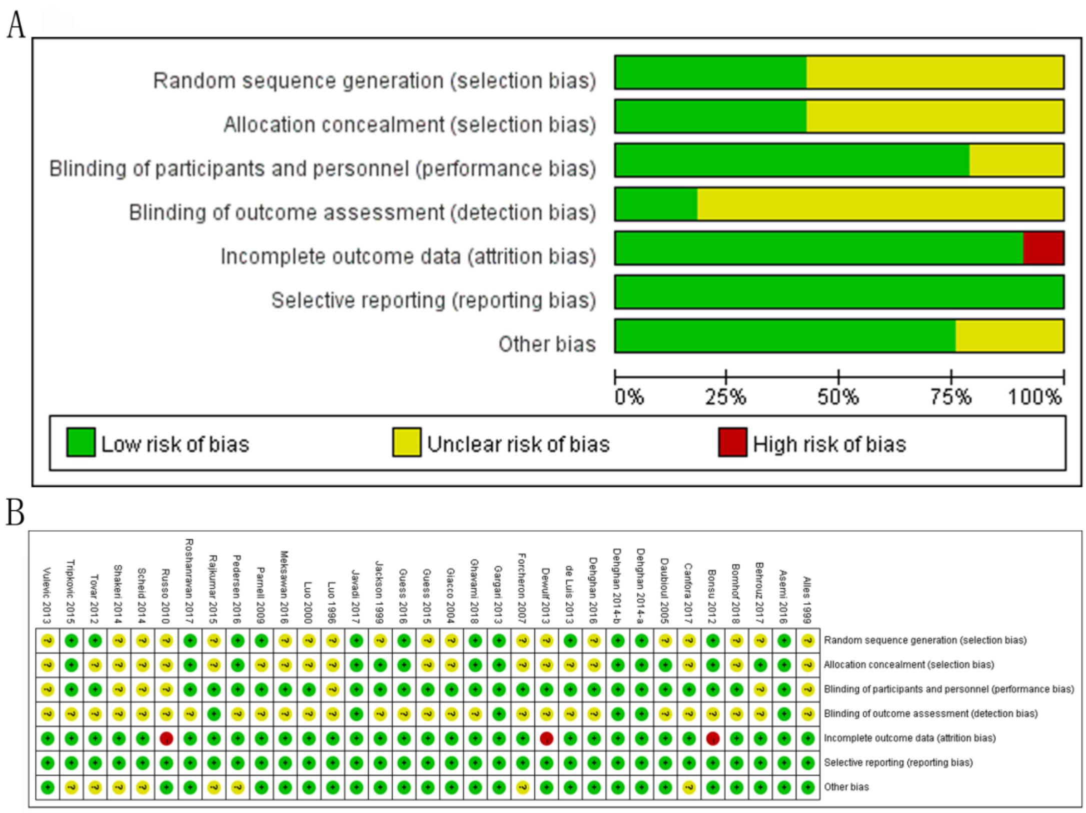

Additional file 2: Figure S1. Risk of bias graph (A) and risk of bias summary (B) in 33 randomized controlled trials
