# Supplementary material for: Inulin-type fructans supplementation improves glycemic control for the prediabetes and type 2 diabetes populations: results from a GRADE-assessed systematic review and dose–response meta-analysis of 33 randomized controlled trials
Source: J Transl Med. 2019 Dec 5;17:410. doi: 10.1186/s12967-019-02159-0 (PMC6896694; doi:10.1186/s12967-019-02159-0)
Supplement: Supplementary file 3 — Additional file 3: Figure S2. Non-linear dose-response analysis between ITF supplement (dose, duration, and total dosage) and glycemic parameters (FBG, HbA1c, FINS, HOMA-IR) levels in the total population. The dose-response analysis was conducted using the nonlinear robust error meta-regression (REMR) model, which is mainly based on the inverse variance-weighted least squares regression and cluster robust error variances for dealing with the synthesis of correlated dose-response data from different studies. The solid line represents weighted mean difference and the dotted lines represent the 95% confidence intervals (CIs). Abbreviations: FBG, fasting blood glucose; FINS, fasting insulin; HbA1c, glycosylated hemoglobin; HOMA-IR, homeostasis model assessment-insulin resistance; T2DM, type2 diabetes mellitus. [file 12967_2019_2159_MOESM3_ESM.pdf]

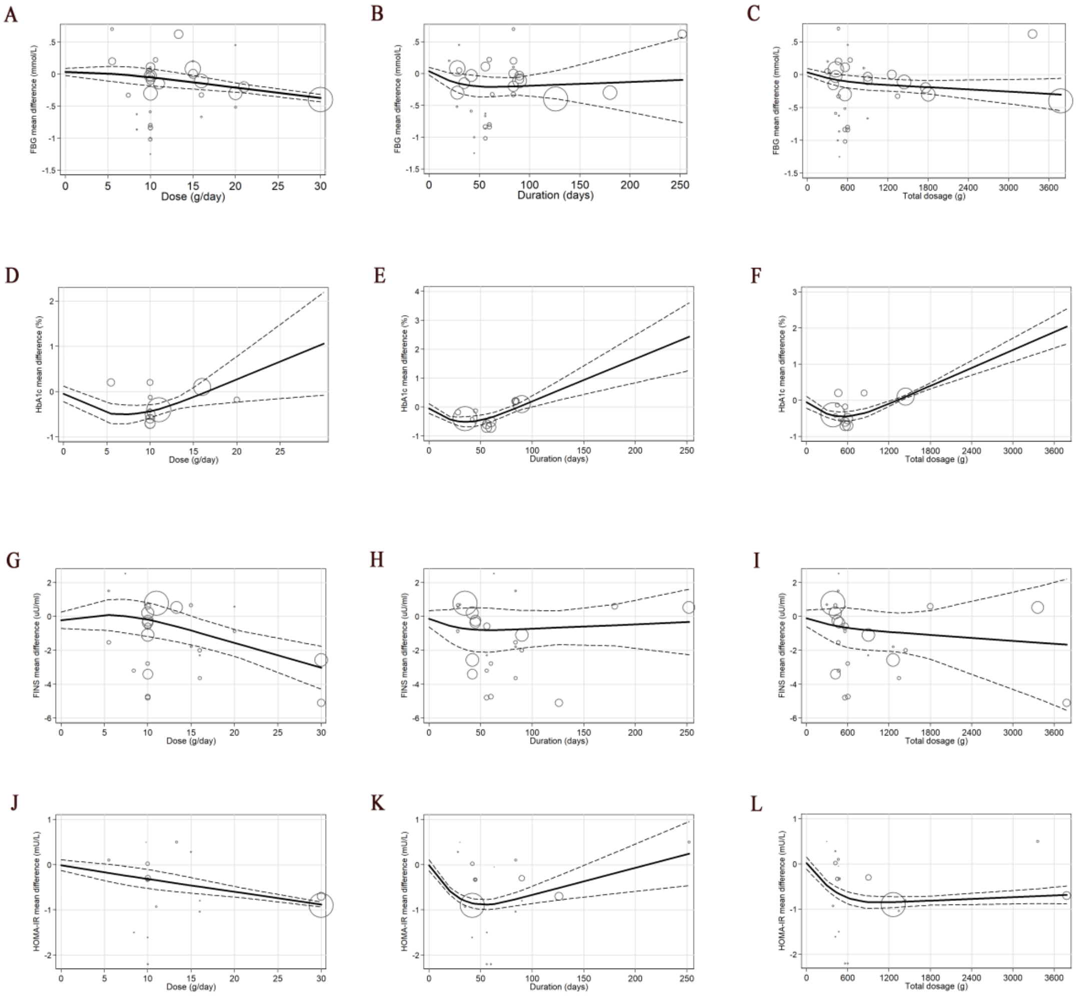

Additional file 3: Figure S2. Non-linear dose-response analysis between ITF supplement (dose, duration, and total dosage) and glycemic parameters (FBG, HbA1c, FINS, HOMA-IR) levels in the total population. The dose-response analysis was conducted using the nonlinear robust error meta-regression (REMR) model, which is mainly based on the inverse variance-weighted least squares regression and cluster robust error variances for dealing with the synthesis of correlated dose-response data from different studies. The solid line represents weighted mean difference and the dotted lines represent the 95% confidence intervals (CIs). Abbreviations: FBG, fasting blood glucose; FINS, fasting insulin; HbA1c, glycosylated hemoglobin; HOMA-IR, homeostasis model assessment- insulin resistance; T2DM, type 2 diabetes mellitus
