# Supplementary material for: Inulin-type fructans supplementation improves glycemic control for the prediabetes and type 2 diabetes populations: results from a GRADE-assessed systematic review and dose–response meta-analysis of 33 randomized controlled trials
Source: J Transl Med. 2019 Dec 5;17:410. doi: 10.1186/s12967-019-02159-0 (PMC6896694; doi:10.1186/s12967-019-02159-0)
Supplement: Supplementary file 5 — Additional file 5: Figure S4. Funnel plots for meta-analysis of inulin-type fructans onFBG (A), HbA1c (B), FINS (C), and HOMA-IR (D). FBG, fasting blood glucose; FINS, fasting insulin; HbA1c, glycosylated hemoglobin; HOMA-IR, homeostasis model assessment-insulin resistance. [file 12967_2019_2159_MOESM5_ESM.pdf]

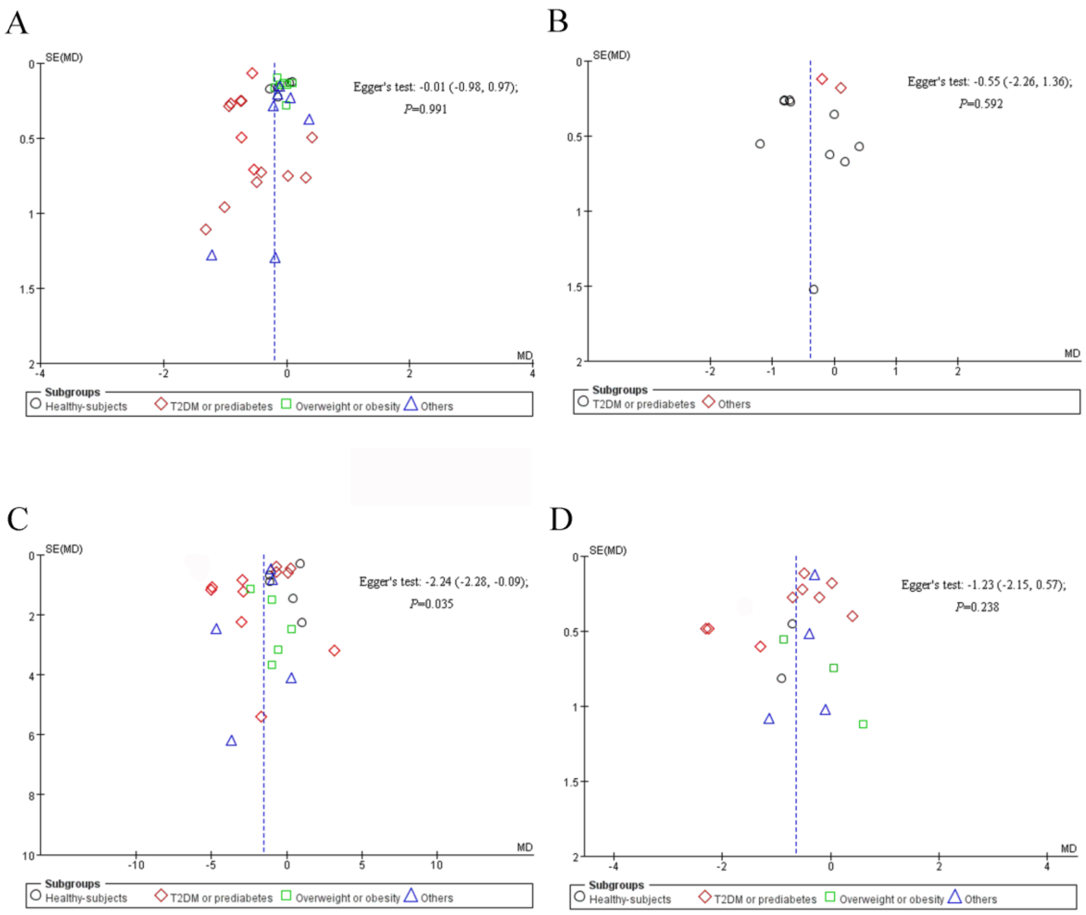

Additional file 5: Figure S4. Funnel plots for meta-analysis of inulin-type fructans on FBG (A), HbA1c (B), FINS (C), and HOMA-IR (D). FBG, fasting blood glucose; FINS, fasting insulin; HbA1c, glycosylated hemoglobin; HOMA-IR, homeostasis model assessment-insulin resistance
